# Osseointegration effects of local release of strontium ranelate from implant surfaces in rats

**DOI:** 10.1007/s10856-019-6314-y

**Published:** 2019-10-12

**Authors:** Ali Alenezi, Silvia Galli, Saba Atefyekta, Martin Andersson, Ann Wennerberg

**Affiliations:** 1 Department of Prosthodontics, Faculty of Odontology, Malmö University, Malmö, Sweden; 2 Department of Prosthodontics, College of Dentistry, Qassim University, Buraidah, Saudi Arabia; 3 Department of Chemistry and Chemical Engineering, Chalmers University of Technology, Göteborg, Sweden; 4 Department of Prosthodontics/Dental Materials Science, Institute of Odontology, Sahlgrenska Academy, University of Gothenburg, Göteborg, Sweden

## Abstract

**Background:**

Numerous studies have reported the beneficial effects of strontium on bone growth, particularly by stimulating osteoblast proliferation and differentiation. Thus, strontium release around implants has been suggested as one possible strategy to enhance implant osseointegration.

**Aim:**

This study aimed to evaluate whether the local release of strontium ranelate (Sr-ranelate) from implants coated with mesoporous titania could improve bone formation around implants in an animal model.

**Materials and methods:**

Mesoporous titania (MT) thin coatings were formed utilizing the evaporation induced self-assembly (EISA) method using Pluronic (P123) with or without the addition of poly propylene glycol (PPG) to create materials with two different pore sizes. The MT was deposited on disks and mini-screws, both made of cp Ti grade IV. Scanning electron microscopy (SEM) was performed to characterize the MT using a Leo Ultra55 FEG instrument (Zeiss, Oberkochen, Germany). The MT was loaded with Sr-ranelate using soaking and the drug uptake and release kinetics to and from the surfaces were evaluated using quartz crystal microbalance with dissipation monitoring (QCM-D) utilizing a Q-sense E4 instrument. For the in vivo experiment, 24 adult rats were analyzed at two time points of implant healing (2 and 6 weeks). Titanium implants shaped as mini screws were coated with MT films and divided into two groups; supplied with Sr-ranelate (test group) and without Sr-ranelate (control group). Four implants (both test and control) were inserted in the tibia of each rat. The in vivo study was evaluated using histomorphometric analyses of the implant/bone interphase using optical microscopy.

**Results:**

SEM images showed the successful formation of evenly distributed MT films covering the entire surface with pore sizes of 6 and 7.2 nm, respectively. The QCM-D analysis revealed an absorption of 3300 ng/cm^2^ of Sr-ranelate on the 7.2 nm MT, which was about 3 times more than the observed amount on the 6 nm MT (1200 ng/cm^2^). Both groups showed sustained release of Sr-ranelate from MT coated disks. The histomorphometric analysis revealed no significant differences in bone implant contact (BIC) and bone area (BA) between the implants with Sr-ranelate and implants in the control groups after 2 and 6 weeks of healing (BIC with a *p*-value of 0.43 after 2 weeks and 0.172 after 6 weeks; BA with a *p*-value of 0.503 after 2 weeks, and 0.088 after 6 weeks). The mean BIC and BA values within the same group showed significant increase among all groups between 2 and 6 weeks.

**Conclusion:**

This study could not confirm any positive effects of Sr-ranelate on implant osseointegration.

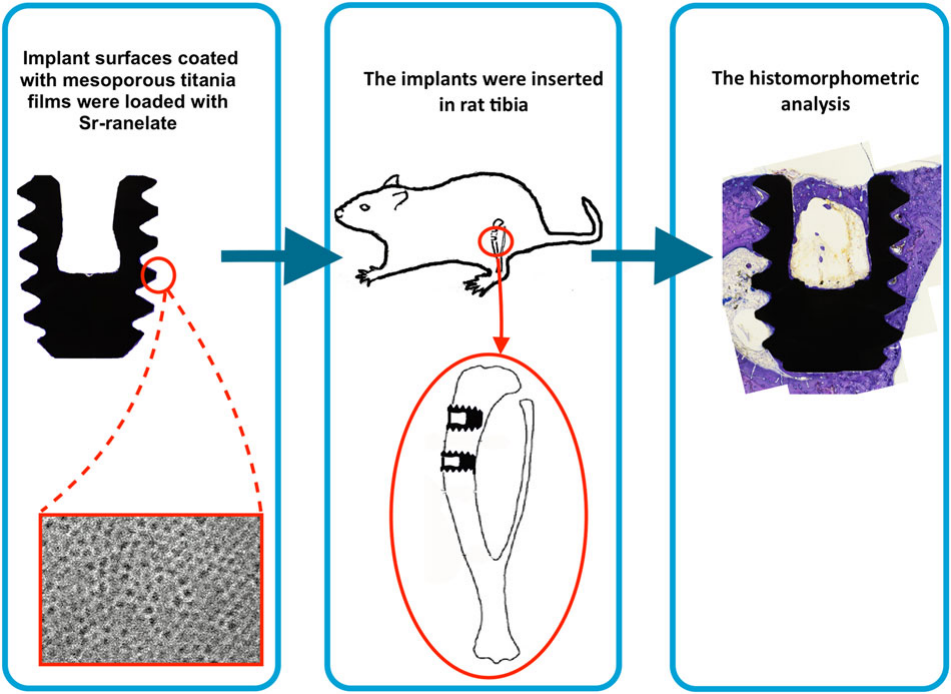

## Introduction

Treatments using dental implants are widely used and have been recognized as a valuable option for restoring missing teeth [1]. Every year, approximately 10 million dental implants are inserted for oral rehabilitation, with a high success rate overall [2]. In some reports, titanium implants have been associated with success rates of more than 95% [3, 4]. In general, the success rate is dependent on numerous factors, such as; the surgical technique, the bone condition, the implant material, the patient conditions, and the performance of the surgical team [5].

Following implant surgery, there are different stages in the healing process before bone maturation is reached, starting shortly after the surgery and continuing for months or years afterwards [6]. Challenges remain in treating conditions associated with poor bone quality or an impaired healing process. Thus, ensuring good implant stability is crucial for implant survival.

To improve osseointegration, many strategies have emerged focusing on implant surface modifications [7]. For example, the implant surface roughness on the micrometer level has shown to have a large impact, and it has been observed that a moderately rough surface demonstrates favorable clinical results [8]. A so increased surface roughness is reported to enlarge the implant surface area, which may increase bone-implant contact [9]; which may result in an improved biomechanical interlocking of the implants in bone, thus be one explanation of the improved clinical results [10].

To further improve implants, surface modifications at the nano level has been suggested [11, 12]. The mechanisms of action induced by these nanofeatures are still not fully understood, but numerous studies have reported that nanofeatures can influence the bone healing process in a positive manner [13]. Nanofeatures are believed to effect protein adsorption and thereby affect cell adhesion and alter cell shape [14, 15]. Furthermore, nanostructured surfaces might facilitate calcium phosphate precipitation, which may accelerate the early stability of implants [16–18]. For instance, in some animal experiments, greater bone formation has been observed around titanium implants coated with CaP nanoparticles compared with pure titanium implants [19, 20].

Mesoporous coatings, having nanostructures in the arrangement of well-ordered pores in the size regime of 2–50 nm, have recently been suggested as a promising surface modification of implants. Such coatings are typically characterized by a homogenously distributed porous structure with high drug-loading capacity, which allows them to be used as a host for drugs and other chemical substances [21, 22]. Some studies have reported a sustained release profile of drugs using implants with a mesoporous coating [21, 23]. The release rate can be controlled by modifying the pore width and surface chemical properties [24].

In dental implant research, the release of drugs from a mesoporous implant coating is primarily used to enhance bone regeneration [25, 26]. Galli et al. [27], for example, evaluated titanium implants coated with thin mesoporous TiO_2_ films loaded with magnesium in rabbit bone. After three weeks, local release of magnesium was associated with greater bone formation at the implant site. In another study, Ti implants coated with 200-nm thick mesoporous films were loaded with osteoporosis drugs (raloxifene and alendronate) and examined in rats [21]. After four weeks, both drugs were associated with significantly improved bone-implant fixation.

Strontium is one possible drug candidate proposed to improve osseointegration of implants [28, 29]. Interestingly, strontium has shown similar biological effects as calcium and is also known for its anti-osteoporotic properties [30]. Thus, some studies have investigated the effects of strontium release on bone formation around implants [28, 31, 32]. Strontium administration was reported to have a positive influence on bone metabolism [33, 34]. It was believed that strontium has dual effects in improving bone formation: stimulating osteoblastic cell proliferation and differentiation and decreasing the activity of osteoclasts [33, 35].

Some in vitro studies have reported an increase in osteoblast attachment and proliferation of calcium phosphate bone substitutes with the incorporation of strontium [36, 37]. Strontium-containing hydroxyapatite (Sr-HA) bone cement was evaluated in revision hip replacement study in goats [38]. Subsequently, after nine months of implantation, the use of Sr-HA revealed good bioactivity and strong bone-bonding ability. Hence, the administration of strontium is assumed to be an effective strategy for improving bone formation around implants. With conventional oral administration of strontium, increased bone levels has been observed when given to dialysis patients [39]. However, some side effects were reported with high oral doses of strontium, such as bone hypomineralization and drug rash with eosinophilia [40–42].

Alternatively, local administration of strontium has been of interest to enhance implant osseointegration while minimizing the risk of potential adverse side reactions. Many researchers have investigated the incorporation of strontium into an implant surface using different methods. For example, surfaces consisting of Titanium/Sr nanotubes have been evaluated and it was shown that incorporation of strontium within the nanotubes enhanced the proliferation of mesenchymal stem cells and osteoblastic differentiation in rats [43]. Park et al. [29] evaluated Ti–6Al–4V alloy implants incorporated with strontium ions produced using a hydrothermal treatment. The implants were inserted in tibial and femoral condyles of rabbits. Four weeks after implantation, implants containing strontium showed significantly more bone-implant contact in both cortical and cancellous bone. In another experiment using rats, Ti implants were coated with hydroxyapatite, with or without strontium, using a sol–gel dip-coating method [44]. Implants dip-coated with strontium showed significantly higher bone formation and stronger fixation compared to implants without this chemical element. In addition, strontium ranelate was examined in an osteoporotic animal model and revealed maintained bone formation level, which eventually prevented trabecular bone loss [45].

Despite the numerous studies showing positive results owing to local administration of strontium on ossoeintegration of implants, it is difficult to determine the true therapeutic effect of strontium. In all above mention examples, strontium has been chemically included within the implant or implant coating, either together with titanium or calcium phosphate, thus inevitably affecting the topography, morphology, chemistry and solubility of the implant surface, which all are known factors affecting ossoeintegration. This circumstance causes it challenging to evaluate the effect of strontium alone, since controls not including strontium are different on several instances.

This present study aimed to evaluate whether the release of Sr-ranelate from an implant surface coated with mesoporous TiO_2_ films could improve bone formation around implants in an animal model. One advantage with this drug-delivery technology is that the only difference between test and control is the presence of strontium, hence the effect that strontium has on ossoeintegration without impact of other factors can be assessed.

## Materials and methods

### Materials

Sr-ranelate was purchased from Sigma–Aldrich (Germany). Pluronic P123 (tri-block copolymer of (ethylene glycol)_20_–(pro-pylene glycol)_70_–(ethylene glycol)_20_), titanium (IV) tetraethoxide, and hydrochloric acid were purchased from Sigma–Aldrich (Germany). Ethanol (99.5%) was provided by Solveco AB (Sweden). Milli-Q water, having an ultrapure grade (18.2 MΩ) was used for all preparations. Titanium QCM-D sensors (QSX 310, Q-sense) were used for the drug uptake and release tests. For the in vivo study, 96 Mini-screws made of titanium grade IV (Neodent, Curitiba, Brazil), with 2.5 mm length and 1.5 mm diameter were used.

### Material preparation

Mesoporous TiO_2_ (MT) films were deposited on both QCM-D sensors and implant mini-screws using the evaporation induced self-assembly (EISA) method, as previously described [46]. In brief, 2.1 g of titanium (IV) tetraethoxide was mixed with 1.6 g concentrated hydrochloric acid (37%) to form a titania precursor solution. The mixture was stirred vigorously until a homogeneous solution was formed. Then, 0.5 g of P123 was dissolved in 8.5 g ethanol with vigorous stirring followed by mixing with the precursor solution. Another solution was prepared simultaneously following the same techniques but with the addition of poly propylene glycol (PPG) to P123 (1:1 by weight) to enable the formation of larger pores [47]. The aim was to form MT having pores of 6 nm (P123) and 7.2 nm (P123 + PPG), in accordance with previous work [24].

The solutions were stirred gently for approximately 24 h. The next day, spin coating was performed by applying 80 µL of the mesporous titania precursor solutions at 4000 rpm for 60 s. Subsequently, the coated surfaces were left for 1 day at room temperature to complete the self-assembly process. The next day, the coated surfaces were calcined by heating with a heating ramp of 1 °C/min from RT to 350 °C. Then, the coated surfaces were left at 350 °C for 4 h to remove the template and to cross-link the titania. For the drug loading, MT coated-implants were soaked in a solution of strontium ranelate dissolved in Milli Q water (0.8 mg/ml) and kept there for one day. Afterward, they were removed and dried gently using nitrogen gas.

### Scanning electron microscopy (SEM)

Scanning electron microscopy was used to visualize the surface morphology of the MT films. A Leo Ultra55 FEG Instrument (Zeiss, Oberkochen, Germany) was used with an accelerating voltage of 2–5 kV. An in-lens secondary electron detector was utilized for top view visualization of the coated surfaces.

### Drug absorption and release

The uptake and release of Sr-ranelate from the MT surfaces were investigated using QCM-D (Q-sense E4). Titanium QCM-D disks were coated with mesoporous TiO_2_ made using P123 or P123-PPG as templates. Uncoated QCM-D disks were used as control. For the experiments, Sr-ranelate dissolved in Milli-Q H_2_O (1 wt% strontium) was first introduced onto the surfaces, to monitor the absorption, and then exchanged with Milli-Q H_2_O, to monitor the release. The experiments were performed under a constant flow of 50 mL min^−1^ and at RT. The observed change in frequency (∆*f*) was used to calculate the change in mass (∆*m*) of the adsorbed Sr-ranelate (ng cm^−2^) using the Sauerbrey equation [48]:

$$\Delta m = - \frac{{C\,\times\,\Delta {\mathrm{f}}}}{n}$$

where*, C* refer to the mass sensitivity constant (17.7 ng Hz^−1^ cm^−2^) and n is the overtone number [49]. Using the calculated mass, the amount of Sr-ranelate absorption and release could be followed as a function of time.

### Animals and surgical procedures

Twenty-four 6-month-old Sprague Dawley female rats were included in this study. The animal surgery was performed under the approval from the ethical committee for animal experiments at the Ecole Nationelle Veterinaire D’Alfort, Masion D’Alfort, France.

At the day of surgery, the rats were anesthetized through inhalation of isoflurane 1% dissolved in O_2_. Then, an incision was made in the skin over the medial face of the tibia. A full-thickness periosteal flap was elevated, and the medial tibia plate was exposed. The implant site was prepared using a sequence of 1-mm- and 2-mm-diameter burs, under constant irrigation of saline solution.

The mini-screws coated with MT were placed in both sides randomly following four experimental groups:

Group 1: mini-screws coated with 6 nm MT.

Group 2: mini-screws coated with 7.2 nm MT.

Group 3: mini-screws coated with 6 nm MT and loaded with Sr-ranelate.

Group 4: mini-screws coated with 7.2 nm MT and loaded with Sr-ranelate.

**Fig. 1:**
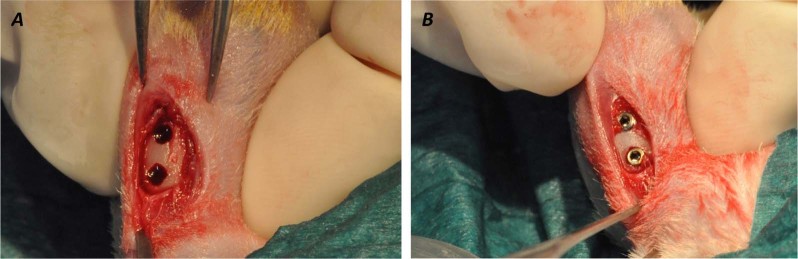
Descriptive surgical procedures: **a** A flap was raised and the implant site was prepared using a sequence of 1-mm- and 2-mm-diameter burs. **b** Implants placement with 2 mm distance between the screws

Each rat received screws in the tibia on both sides, with a total of four screws per animal, two tests and two controls (Fig. 1).

After two and six weeks of healing, the rats were sacrificed by pentobarbital overdose. The implants with the surrounding bone were removed from the tibia and fixed in 70% ethanol for histological processing.

### Histological analysis

A total number of 96 implants were retrieved with the surrounding bone and processed for histological analyses (12 per group for each time point). The retrieved bone blocks were processed in series of dehydrations in ethanol and immersed in light-curing resin (Technovit 7200 VLC—Heraeus Kulzer, Wehrheim, Germany) and finally embedded in the resin. Thereafter, the resin-embedded samples were subjected to undecalcified ground sectioning using Exakt sawing and grinding equipment [50]. The sections were ground to a final thickness of around 20 µm and histologically stained with Toluidine blue-pyronin dye. For histological evaluations, the stained sections were examined using an optical microscope (Eclipse ME600—Nikon Co., Tokyo, Japan). For histomorphometry, the following parameters were calculated; the percentage of bone-implant contact (BIC), the percentage of bone area between threads (BA), and the percentage of new bone area (new-BA) between threads. The histomorphometric analysis was performed using the Image J software (National Institutes of Health, Bethesda, MD, USA).

### Statistical analysis

The non-parametrical Kruskal–Wallis test (SPSS Statistics v. 22, IBM Corp., USA) was used to evaluate the differences between all groups in the animal experiment. Mann-Whitney U test was used to compare the histomorphometrical values within same group after 2 and 6 weeks. The significance level was set at *P* = 0.05. All data were plotted as mean ± standard deviation.

## Results

### Surface evaluation

**Fig. 2:**
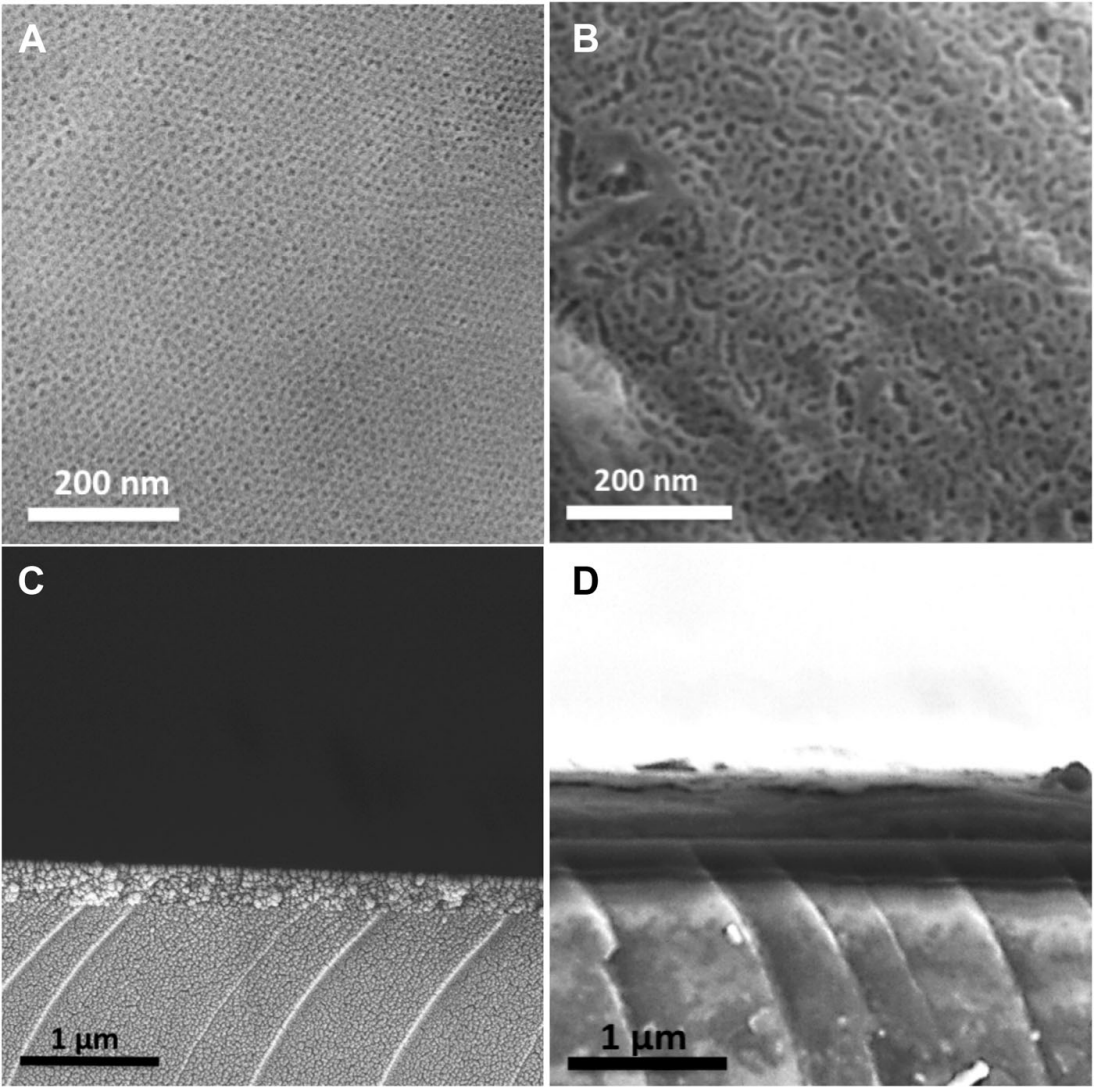
SEM micrographs showing: **a** a top view of MT thin film formed using P123 as template, **b** a top view of MT thin film formed using P123 + PPG as template, **c** a cross-sectional view of MT thin film formed using P123 as template, and **d** a cross-sectional view of MT thin film formed using P123 + PPG as template

SEM evaluation revealed a well-ordered porous structure of the two different MT films formed on titanium disks (Fig. 2). The measured pore size was 6 nm for the film synthesized with P123, and 7.2 nm for the film synthesized by adding PPG to P123. Images of the film cross-sections revealed film thicknesses of approximately 300 nm (P123) and 750 nm (P123 and PPG), Fig. 2. The observed difference in the MT thickness is believed to be a result of change in the solution viscosity caused by the addition of PPG.

### Drug absorption and release rate

**Fig. 3:**
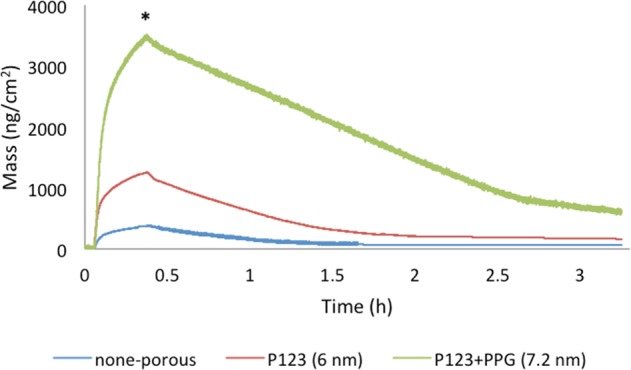
QCM-D results showing Sr-ranelate absorption and release from MT films. The asterisk (*) indicates the time when Sr-ranelate solution was exchanged with flow of Milli-Q H_2_O

Quartz crystal microbalance with dissipation monitoring (QCM-D) was used to study the absorption and release behavior of Sr-ranelate from the two MT surfaces, that is, with 6 and 7.2 nm pore sizes, as shown in Fig. 3. The flow rate of 50 mL/min used in this test demonstrate an accelerated condition compared to the in vivo situation in bone tissue. The differences in pore size and film thickness of the two films were shown to highly affect the loading capacity and release of Sr-ranelate. Using the Sauerbrey equation, the analysis revealed an absorption maximum of 3300 ng/cm^2^ of Sr-ranelate on the 7.2 nm MT, which was about 3 times more than the observed amount on the 6 nm MT (1200 ng/cm^2^). Both groups showed similar release kinetics in which an initial fast release was observed followed by sustained release of Sr-ranelate after solution exchanged with Milli-Q water (Fig. 3).

### In vivo experimentation

No rats died during the surgery or during the healing period and all the animals healed uneventfully. No signs of infection or disabilities in motion were observed.

### Histological results

The qualitative evaluations of the histological sections showed that deeply stained woven bone had formed around the surface of all implants after 2 weeks of healing. After 6 weeks, more new bone had formed around the surface of all implants filling the area between the threads. Overall, both evaluated surfaces showed similar bone response after 2 and 6 weeks.

**Fig. 4:**
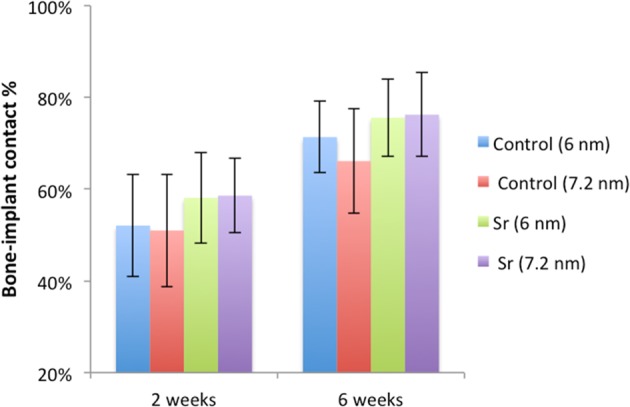
Bone-implant contact percentage along threads. *P* value = 0.43 at 2 weeks and 0.172 at 6 weeks

**Fig. 5:**
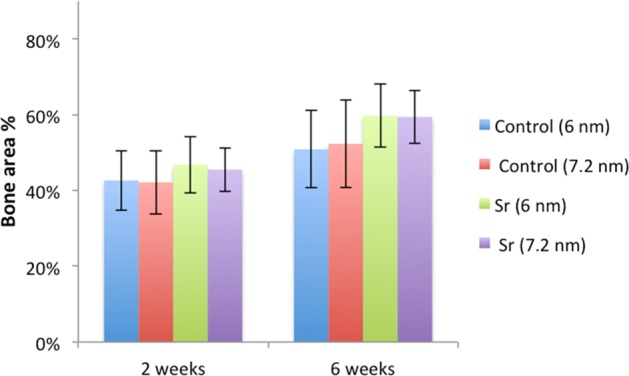
Bone area percentage between threads. *P* value = 0.503 at 2 weeks and 0.088 at 6 weeks

**Fig. 6:**
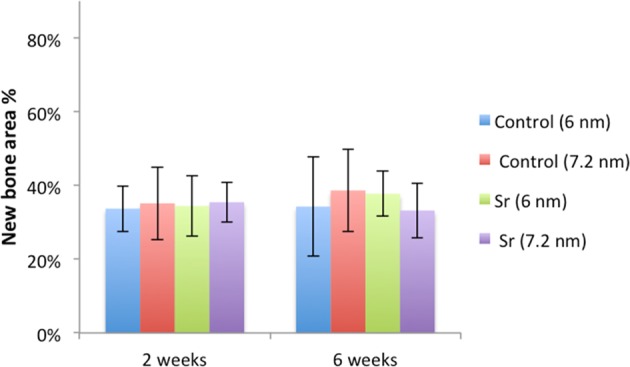
Percentage of new bone area between the threads. *P* value = 0.78 at 2 weeks and 0.464 at 6 weeks

**Fig. 7:**
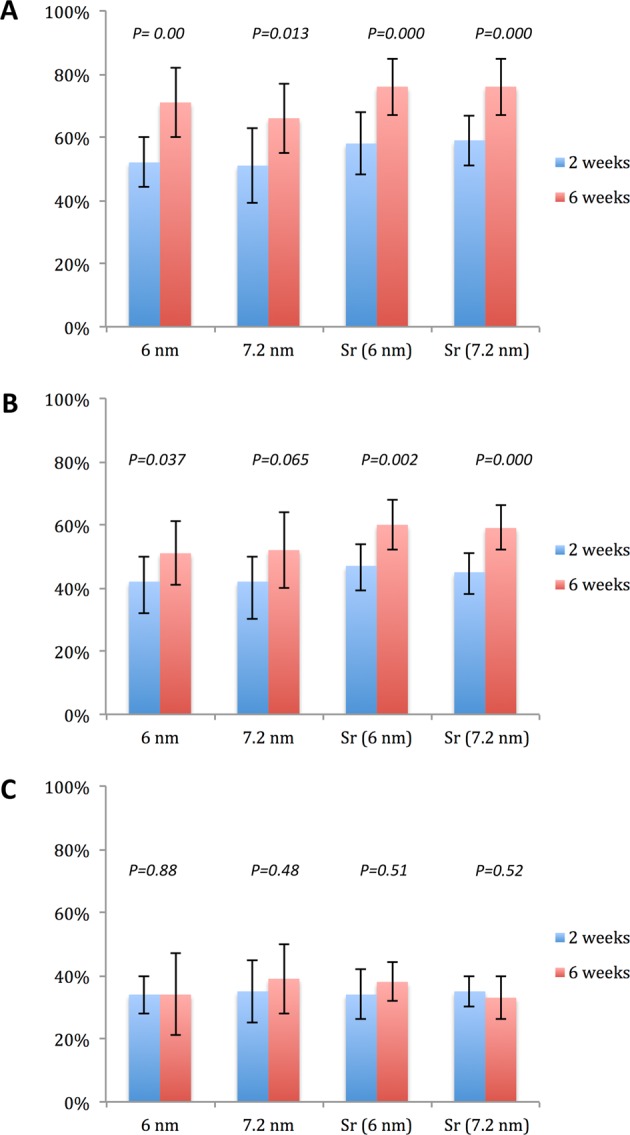
Histomorphometrical values; BIC% **a**, BA% **b**, and new-BA% **c**, within the same group between 2 and 6 weeks. The significance level was set at *P* = 0.05

**Fig. 8:**
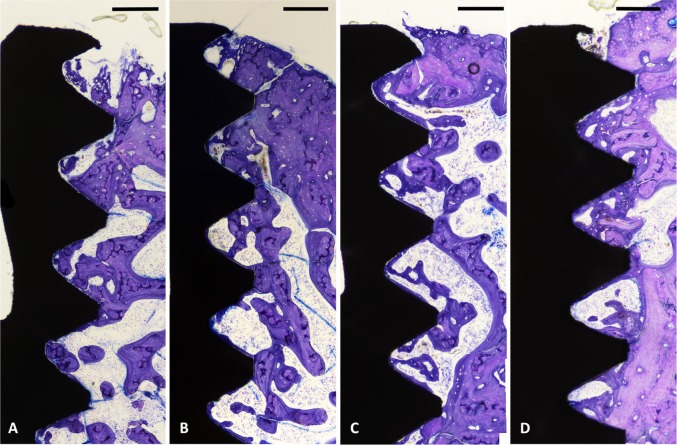
Histological micrographs obtained after 2 weeks of healing for implants coated with MT: **a** 6 nm, **b** 7.2 nm, **c** 6 nm loaded with Sr-ranelate, and **d** 7.2 nm loaded with Sr-ranelate. Scale bar is 200 µm

**Fig. 9:**
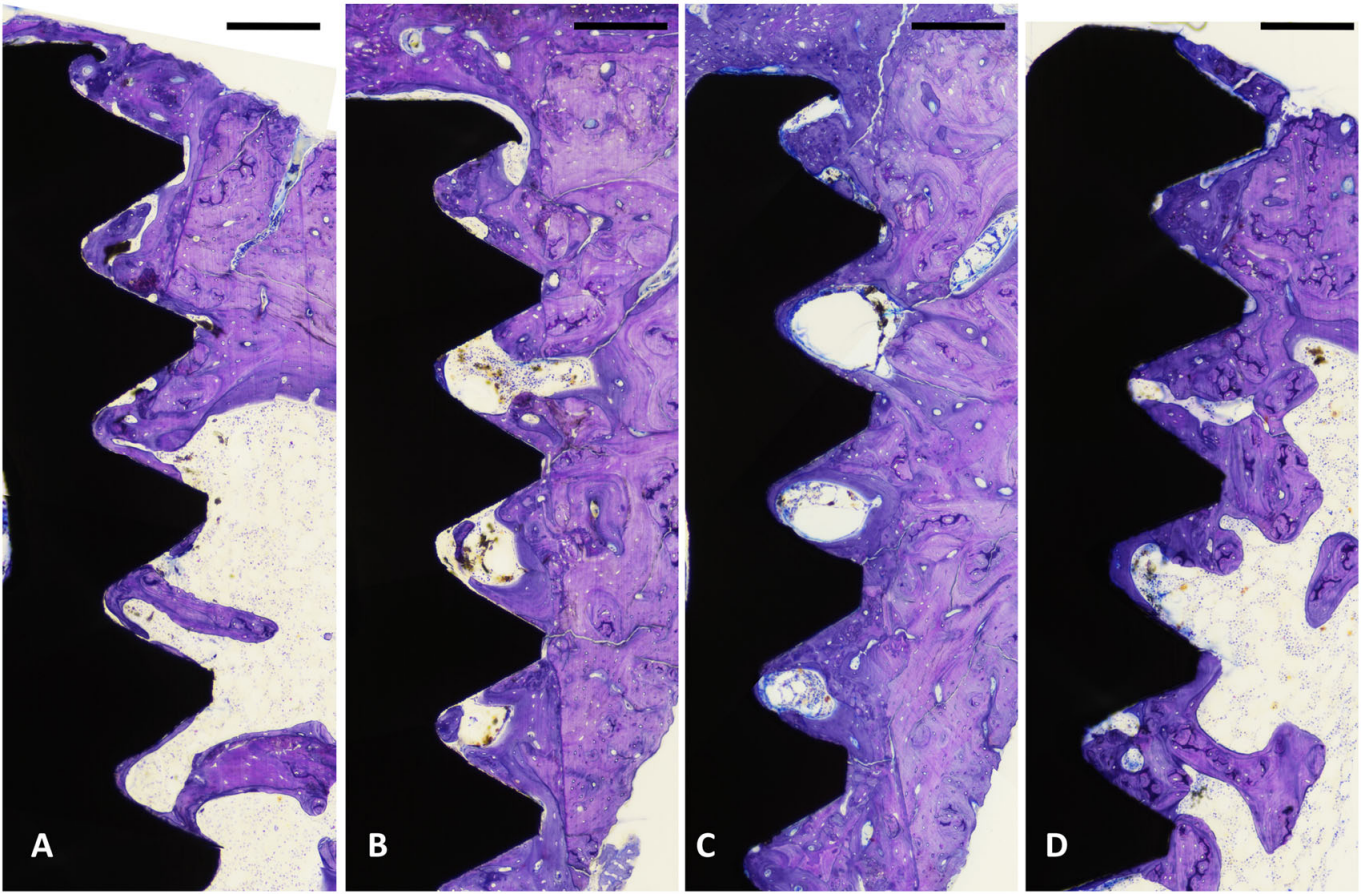
Histological micrographs obtained after 6 weeks of healing for implants coated with mesoporous films: **a** 6 nm, **b** 7.2 nm, **c** 6 nm loaded with Sr-ranelate, and **d** 7.2 nm loaded with Sr-ranelate. Scale bar is 200 µm

**Fig. 10:**
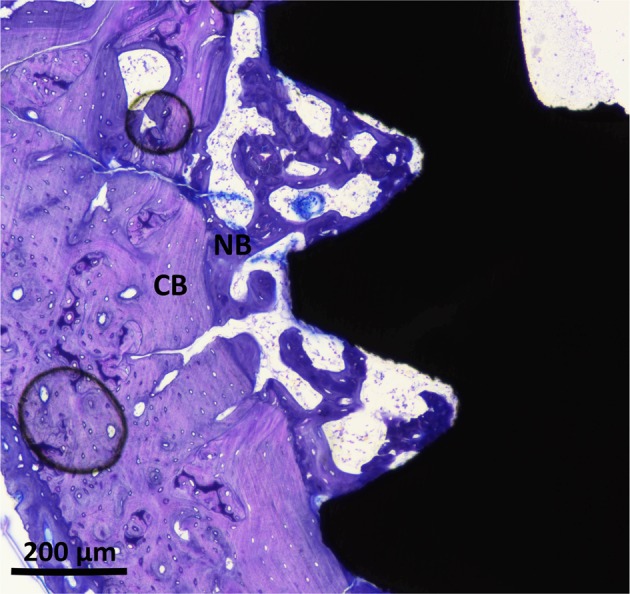
Histological micrograph at high magnification for early bone formation around implants coated with MT, 6 nm, after 2 weeks of healing. New bone starts to fill the areas between cortical bone and implant. Cortical bone (CB) is visualized in pale red while the New Bone (NB) is visualized in purple

**Fig. 11:**
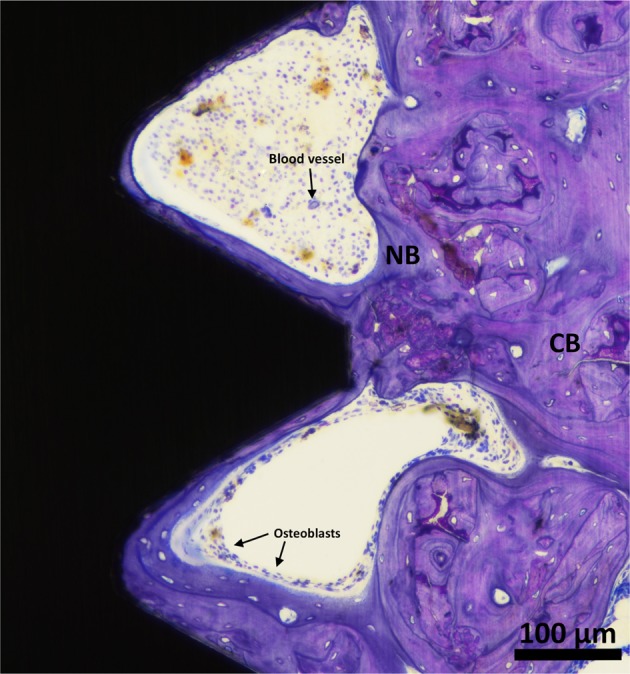
Histological micrograph at high magnification for formed bone along the threads for the screw coated with MT, 6 nm, and loaded with Sr-ranelate after 6 weeks of healing time. Large number of osteoblasts can be seen over the formed bone layer to help build new bone. Cortical bone (CB) is visualized in pale red while New Bone (NB) is visualized in purple

The histomorphometric analyses for BIC, BA, and new-BA are summarized in Table 1 and Figs 4–7. After two and six weeks, higher percentages of BIC and BA observed around screws incorporated with Sr-ranelate (Figs 8–11). Layers of newly formed bone lining the implant threads characterized many of the implants incorporated with Sr-ranelate (Fig. 11). In addition, large remodeling lacunae with osteoblasts were visible at this area. However, the statistical analysis revealed that the differences between control and test groups were not significant. For BIC, the *P* value was 0.430 at 2 weeks and 0.172 at 6 weeks. For BA, the *P* value was 0.503 at 2 weeks and 0.088 at 6 weeks. The mean BIC and BA values within the same group showed significant increase among all groups between 2 and 6 weeks (Fig. 7).

**Table 1:** Summary of the histomorphometric measurements

	BIC % (SD)		BA % (SD)		New-BA % (SD)	
Group	2 weeks	6 weeks	2 weeks	6 weeks	2 weeks	6 weeks
Control (6 nm)	52 (11)	71 (8)	42 (8)	51 (10)	34 (6)	34 (13)
Control (7.2 nm)	51 (12)	66 (11)	42 (8)	52 (12)	35 (10)	39 (11)
Sr (6 nm)	58 (10)	76 (9)	47 (7)	60 (8)	34 (8)	38 (6)
Sr (7.2 nm)	59 (8)	76 (9)	45 (6)	59 (7)	35 (5)	33 (7)

## Discussion

Several studies have claimed that the use of strontium can enhance the formation of new bone and reduce bone resorption [32, 51, 52]. However, systemic administration of strontium might be associated with some unpleasant side effects, such as diarrhea [18]. As an alternative to systemic administration, local drug-delivery at the bone-implant interface has gained attention. Such local administration may be beneficial to patients as it avoids possible side effects and has immediate effects at minimal but efficient concentrations.

So far, most of the methods used for local delivery of strontium to the bone-implant interface have been based on incorporating strontium into the implant surface. Such incorporation may change the surface properties of the implant, such as surface morphology and material chemistry [43, 53, 54]. As a consequence, several factors might have contributed to the observed changes in tissue response, making it impossible to identify if the observations are due to the effect of Sr alone.

Of particular interest to implants research, mesoporous titania (MT) loaded with bone-stimulating agents can be used to enhance implant osseointegration. By changing the surface characteristics of the MT films, the absorption and release behavior of the loaded substance can be altered. This property was proven in this work, as the difference in pore size changed the Sr-renalate absorption and release rates significantly. MT with a 7.2 nm pore size demonstrated almost triple the loading capacity compared to mesoporous Ti with a 6 nm pore size. Since the 7.2 nm MT films was 2 times thicker than the 6 nm MT films, also the pore size had an effect on the capacity. While the clinical therapeutic effect of the released drug is directly linked to the dose, further optimization of both pore size and film thickness can be performed for optimal effect. In addition, the drug-loading capacity and kinetics can be further controlled by altering and surface chemistry [55, 56].

Cecchinato et al. [57] investigated in vitro the absorption and release of magnesium from MT implant coatings with pore sizes similar to the ones used in this study. They found higher amount of magnesium absorbed into the MT coating with 7 nm (450 ng/cm^2^) compared to 6 nm (300 ng/cm^2^). Our QCM-D results demonstrated higher amount of immobilized Sr compared to what have been reported with magnesium. When the projected screw surface area, 0.2 cm^2^, is taken into account this corresponds to 660 ng (7.2 nm) and 240 ng (6 nm) Sr-renalate loading into the MT coatings per implant. If we then assume a diffusion distance of 1 mm, the distribution volume is at the order of 0.1 cm^3^. The resulting Sr-renalate concentrations become 6.6 µg/ml (7.2 nm) and 2.4 µg/ml (6 nm). These Sr concentrations are within the optimized values of 0.21 and 21.07 µg/ml of Sr ranelate that has been shown to induce mesenchymal stem cells to differentiate into osteoblasts as determined in a previous in vitro study by Sila-Asna et al. [35]. The authors further showed that high concentrations of up to 210.7 µg/ml might be associated with delayed effects on osteoblastic differentiation. Other studies have reported that the effect of strontium on bone is complex and dose-related [58].

In our in vivo experiment, using rats as an animal model, the healing process was observed after two and six weeks to evaluate bone formation around implants with and without Sr-ranelate. Although rat bone have biological dissimilarities to human bone [59], the use of these animals is common in implant research [44]. It should be noted that rats have a shorter healing-time and bone turnover period compared to humans, which facilitates the evaluation of osseointegrated implants at different healing stages within a shorter time frame [60]. The rats were examined at two weeks to assess early bone formation, as the healing process is expected to start shortly before that, and they were examined after six weeks, when the bone formation process is expected to be completed. To accommodate the small bone size of the rats, miniature titanium screws were used in this experiment.

Before surgery, Sr-ranelate was loaded into the MT by simple immersion followed by gentle drying to remove excess solution. It is hypothesized that the addition of Sr-ranelate will not be associated with any change to the surface morphology. Since Sr-renalate is water soluble, hence any Sr-renalate that might be present, if unlikely, on the implant surface would directly dissolve upon contact with blood and a pure MT surface would be revealed. As a direct consequence of this, the use of our MT system makes it possible to directly evaluate the presence of Sr, without the inference of changes in surface topography or implant material chemistry.

For the histomorphometric analyses, parameters such as BIC, BA, and new-BA are commonly used to quantify osseointegration. This is a two-dimensional analysis suitable to directly observe bone formation along implant threads. The results from this study could not demonstrate improved osseointegration for implants containing Sr-ranelate independent if the pore diameter was 6 or 7.2 nm. Even though superior values of BIC and BA were seen for implants containing Sr-ranelate in both early and late healing stages, these values were not significantly different between test and control. Although the beneficial effects of incorporating strontium into implant surfaces have been reported in numerous studies, the absence of significant differences between the control and test implants in this work could be due to the fact that Sr alone has an insignificant effect on osseointegration in healthy animals [29, 61].

Because of its anti-osteoporotic properties, the use of Sr-ranelate in the medical field is mainly for treatment of osteoporosis [18, 34]. This is the central reason for why some of the investigated endoosseous implants incorporated with strontium were performed using osteoporotic animal models [44]. Thus, the effects of strontium on bone might be less evident when using animals with normal bone conditions. Although the positive effects of Sr on implant ossoeintegration in normal bone condition were reported by other authors [29, 32]. Park et al. [29] evaluated in vitro and in vivo Ti–6Al–4V alloy implants incorporated with strontium by the use of a hydrothermal treatment. In their study, the control (untreated) and hydrothermally treated surfaces showed similar surface roughness values; however, surface chemistry was obviously different. Evaluation of cell attachments and percentage of bone area revealed comparable results between test and control surfaces. However, significantly greater removal torque and BIC values were seen for the Ti64/Sr implants.

Small animals like rats have been reported to have great healing capacity, which make the process of detecting differences during bone healing less straightforward [62]. Another explanation for the lack of significant differences between test and control is that all groups showed enhancements in osseointegration related to the presence of the mesoporous films, which have been reported to stimulate cell attachment and differentiation. As previously discussed, MT coatings can increase the surface area and facilitate implant integration with bone [46]. Interestingly, we observed early bone apposition lining the implant threads for groups containing Sr-ranelate (Fig. 11). This could be due to the presence of Sr-ranelate, which probably promoted osteoblastic differentiation.

## Conclusion

In summary, the highly porous structure of mesoporous TiO_2_ film is a suitable host material for drug-delivery applications. This study could not confirm the positive effects of Sr-ranelate on implant incorporation in bone of healthy rats.
